# Triggering of Inflammasome by Aggregated α–Synuclein, an Inflammatory Response in Synucleinopathies

**DOI:** 10.1371/journal.pone.0055375

**Published:** 2013-01-31

**Authors:** Gaia Codolo, Nicoletta Plotegher, Tommaso Pozzobon, Marco Brucale, Isabella Tessari, Luigi Bubacco, Marina de Bernard

**Affiliations:** 1 Department of Biology, University of Padua, Padua, Italy; 2 Venetian Institute of Molecular Medicine, Padua, Italy; 3 National Research Council (CNR), Institute of Nanostructured Materials (ISMN), Montelibretti, Rome, Italy; University of Nebraska Medical Center, United States of America

## Abstract

Parkinson’s disease (PD) is one of the most common neurodegenerative diseases. It is characterized by the loss of dopaminergic neurons in the *substantia nigra pars compacta* of the brain. Another feature is represented by the formation in these cells of inclusions called Lewy bodies (LB), principally constituted by fibrillar α-synuclein (αSyn). This protein is considered a key element in the aetiology of a group of neurodegenerative disorders termed synucleinopathies, which include PD, but the cellular and molecular mechanisms involved are not completely clear. It is established that the inflammatory process plays a crucial role in the pathogenesis and/or progression of PD; moreover, it is known that aggregated αSyn, released by neurons, activates microglia cells to produce pro-inflammatory mediators, such as IL-1β. IL-1β is one of the strongest pro-inflammatory cytokines; it is produced as an inactive mediator, and its maturation and activation requires inflammasome activation. In particular, the NLRP3 inflammasome is activated by a wide variety of stimuli, among which are crystallized and particulate material. In this work, we investigated the possibility that IL-1β production, induced by fibrillar αSyn, is involved the inflammasome activation. We demonstrated the competence of monomeric and fibrillar αSyn to induce synthesis of IL-1β, through TLR2 interaction; we found that the secretion of the mature cytokine was a peculiarity of the fibrillated protein. Moreover, we observed that the secretion of IL-1β involves NLRP3 inflammasome activation. The latter relies on the phagocytosis of fibrillar αSyn, followed by increased ROS production and cathepsin B release into the cytosol. Taken together, our data support the notion that fibrillar αSyn, likely released by neuronal degeneration, acts as an endogenous trigger inducing a strong inflammatory response in PD.

## Introduction

Parkinson’s Disease (PD) is one of the most common neurodegenerative diseases and affects more that 1% of people over the age of 60 years worldwide [1]. It is characterized by death of dopaminergic neurons of the *substantia nigra pars compacta* (SNpc) of the brain [2] and by the presence of intracellular aggregated inclusions containing mainly α-synuclein (αSyn), called Lewy bodies (LB) [3], [4]. The disease can be divided into sporadic and early-onset familial PD; the latter is linked to three missense mutations, A53T, A30P and E46K, as well as multiple copies of the wild-type (*wt*) αSyn gene [5], [6], [7], [8]. Given that αSyn is the major component of LB in both familial and sporadic PD cases, it is considered a critical factor in PD aetiology [4]. Currently, the cellular and molecular mechanisms underlying the pathological actions of αSyn are poorly understood, and the factors contributing to sporadic PD, representing about 90% of PD cases, are not known. Both *in vitro* and *in vivo*, αSyn can aggregate to form oligomeric species and ordered fibrils in LB, characterized by a cross β-sheet structure, that are morphologically similar to the amyloid fibrils found in neuritic plaques in Alzheimeŕs disease, as well as in deposits associated with other amyloidogenic disorders [9]. Although αSyn is typically considered an intracellular protein, it has also been found in extracellular biological fluids, including human cerebrospinal fluid and blood [10], [11], [12], [13], [14]. Changes in the levels and characteristics of extracellular αSyn are associated with the disease [15]. One of the contributing factors to αSyn spread could be the membrane permeability of dying cells, but it has also been suggested that vesicle-mediated exocytosis from normal cells is probably the main source of extracellular αSyn [15]. Furthermore, extracellular αSyn has been shown to be taken up by neuronal and microglial cells in culture, although the nature of the mechanism involved is still controversial [15].

It is established that disease onset and progression are characterized by inflammation and immune abnormalities, including the activation of microglia, but the mechanism and role of this activation remains controversial. The current body of evidence points to aggregated and modified forms of αSyn as a primary cause of microglia activation [16], [17], [18], [19], [20], [21], [22], [23]. Moreover, there is substantial evidence that microglia are activated in mouse, rat and non-human primate models of PD prior to frank neuron death. Collectively, these observations support the hypothesis that αSyn is released early in the disease and, acting as an endogenous disease-related signal, it activates microglia to release pro-inflammatory molecules, such as TNF-α and IL-1β, which are detrimental to dopamine neurons [24], [25]. The progressive death of the latter is expected to be responsible for further αSyn release, thus amplifying the neurodegenerative process [26]. Although this model clearly suggests that αSyn exerts a pivotal role in promoting inflammation by acting on microglia, it remains to be firmly established which form of the protein acts as the triggering signal and through which mechanism.

IL-1β is one of the most abundant pro-inflammatory cytokines that broadly affects inflammatory processes [27]. Its production is tightly controlled: it is synthesized as an inactive pro-protein and its activation and release are controlled by the cysteine protease caspase-1, which cleaves the protein into the active form [28], [29]. Caspase-1 is synthesized as an inactive zymogen (pro-caspase-1) and becomes proteolytically active only after controlled dimerization in multi-protein complexes, called inflammasomes [30], [31]. Inflammasomes are a group of protein complexes that recognize a large variety of inflammation-inducing stimuli that include pathogen-associated molecular patterns (PAMPs) and danger-associated molecular patterns (DAMPs) [32]. Different inflammasome complexes are known; among these, NLRP3 is the most studied due to the large variety of signals that activate it, including LPS, bacterial toxins, particulate materials, amyloid-β and prion protein fibrils [33], [34], [35], [36]. Several models have been proposed to explain how all these heterogeneous signals activate the NLRP3 inflammasome; these non-exclusive mechanisms include both direct and indirect signal recognition mediated by additional accessory proteins [37], [38], [39].

In this work, we studied the pro-inflammatory activity exerted on monocytes by fibrillar and monomeric αSyn, and we found that both promote the expression of pro-IL-1β, following the engagement of the receptor TLR2, but only αSyn fibrils induce the release of the mature form of IL-1β, via inflammasome activation. We show that protein complex activation requires fibril phagocytosis followed by cathepsin B release into the cytosol, but it also relies on the production of ROS.

Collectively, our data provide the evidence that, among the different αSyn forms, fibrils are the main trigger of the inflammatory response, which likely precedes neurodegeneration; our observation might be useful to develop a novel therapeutic avenue for PD.

## Materials and Methods

### Ethics Statement

Peripheral blood mononuclear cells utilized in this study derived from buffy coats obtained from healthy blood donors, as anonymously provided by the Transfusion Centre of the Hospital of Padua. Written informed consent for the use of buffy coats for research purposes was obtained from blood donors by the Transfusion Centre. Data related to human samples were all analyzed anonymously. Human leukocytes, provided by the Transfusion Centre of the Hospital of Padua, were obtained not consequently to experimentation on human beings but as a consequence of voluntary and informed blood donation for transfusions: no approval of Ethics Committee is needed in such cases in our institution.

### Reagents

PBS, RPMI 1640, and FCS were from Euroclone (Siziano, Italy). Gentamicin, HEPES, TRIzol solution, 4–12% SDS-PAGE gels, Hoechst and SuperScript II were from Life Technologies (Glasgow, Scotland, UK). Nutridoma-SP and NP-40 were from Roche Applied Science (Basel, Switzerland). Ac-YVAD-CMK, H_2_DCF-DA and CA-074-Me were from Cayman Chemical (Ann Harbor, MI, USA). Polyclonal antibody anti-caspase-1 was from Upstate (Lake Placid, NY, USA). Bafilomycin A1 (Baf A1), tetramethylbenzidine, LPS, tetramethylrhodamine-5-maleimide (TCEP), aprotinin, leupeptin, PMSF, diphenyleneiodonium chloride (DPI) were obtained from Sigma-Aldrich (St. Louis, MO, USA). Polyclonal antibody against IL-1β was from R&D Systems (Wiesbaden, Germany). Monoclonal antibody against actin was from Calbiochem (La Jolla, CA, USA). Monoclonal blocking antibody anti-TLR2 was from eBiosciences (San Diego, CA, USA). Ficoll-Paque solution, Percoll and ECL (ECL system) were from GE Healthcare (Chalfont St Giles, Buckinghamshire, UK). BCA protein assay reagent was from Pierce (Rockford, IL, USA).

### Expression and Purification of αSyn

Human αSyn and its mutant (αSynC141), suitable for fluorophore labelling via thiol chemistry, were cloned in pET-28a (Novagen, Darmstadt, Germany). The mutated form was obtained by mutagenic PCR, introducing a triplet codifying for cysteine in position 141.

Both proteins were expressed in a lipid A mutant of *E. coli,* BL21(DE3) [40], with strongly reduced endotoxicity (kindly provided by Prof. J.F. Gauchat). Bacteria were grown to an OD of 0.4 at 600 nm and induced with 0.1 mM IPTG for 5 h. Cells were then collected by centrifugation and recombinant proteins recovered from the periplasm by osmotic shock, as previously described [41], [42].

The periplasmic homogenate was then boiled for 10 min and the soluble fraction, containing αSyn, was subjected to a two-step ammonium sulphate precipitation (corresponding, respectively to ammonium sulphate percent saturation of 35% and of 55%). The pellet was then resuspended, dialyzed against 20 mMTris-HCl pH 8.0, loaded onto a 6 ml Resource Q column (GE Healthcare, Chalfont St Giles, Buckinghamshire, UK) and eluted with a 0–500 mM linear gradient of NaCl. Protein was then dialyzed against deionized water, lyophilized and stored at −20°C.

### Fluorescent Labelling of αSyn

Labelling was performed by adding a 2.5-fold molar excess of *tris*(2-carboxyethyl)phosphine (TCEP) to αSynC141, dissolved in PBS, pH 7.4; after 30 min of incubation at room temperature, TCEPwas added to the protein solution in a 5∶1 stoichiometric ratio and the reaction was left at 45°C for 4 h.

The protein was extensively dialyzed to eliminate excess fluorophore and reducing agent; the purity of the conjugated protein was checked by reverse phase chromatography (C4 column, Phenomenex, Torrance, CA, USA).

The absorbance of rhodamine-labelled αSyn was measured at 572 nm with an UV-visible spectrophotometer (Agilent 8453, Santa Clara, CA, USA) and the protein concentration was calculated considering the molar extinction coefficient of the fluorophore (ε_RHOD_ = 95000 M^−1^cm^−1^).

### Monomeric αSyn Preparation and Fibrils Growth

αSyn was resuspended in PBS, pH 7.4, and filtered to remove oligomerized protein with a 0.22 µm cut-off filter. Initial protein concentration was quantified by spectrophotometer (Agilent 8453, Santa Clara, CA, USA), measuring the absorbance at 276 nm.

Before starting the aggregation, the initial monomer concentration was adjusted to 100–200 µM and the solution was supplemented with 0.05% (w/v) sodium azide as bacteriostatic agent. The protein solution was then shaken at 1000 rpm at 37°C for 2 weeks and the fibrils were collected by centrifugation. The pellet was washed several times to remove non fibrillar αSyn and sodium azide, and resuspended in sterile PBS to a final monomer equivalent concentration of about 200 µM. The monomer equivalent concentration in the fibril pellet was calculated by difference between the starting monomer concentration and the residual monomer supernatant concentration after fibrillation. To reach the fibril amount corresponding to a concentration of 200 µM in aggregated monomers, the fibril pellet was resuspended in the proper volume of PBS. Considering the statistical-mechanical model developed to describe fibrils formation [43] and the AFM-assed average fibril length [44], we assumed an average of 5,000 to 10,000 αSyn monomers per fibril, leading to an estimated concentration of fibrils of 40 nM.

Fluorescent fibrils were obtained by adding to the initial protein solution 5% of rhodamine-labelled αSynC141 and following the same procedure.

Non fluorescent and fluorescent fibrils were characterized using transmission electron microscopy, confocal microscopy and fluorescence spectroscopy.

### Thioflavin T (ThT) Assay

ThT assay is typically used to detect amyloid fibrils, since the ThT dye binds to β-sheet rich structures and, when bound, it shows an enhanced fluorescence emission spectrum [45]. ThT binding assays were performed using a filtered (0.22 µm cut-off) 25 µM ThT solution in 25 mM sodium phosphate (pH 7.0). At the end of the aggregation, before dividing pellet and supernatant, an aliquot of protein samples was taken and diluted into the ThT-containing buffer (final volume 500 µl). Fluorescence emission measurements were conducted on a fluorimeter (Ls50, Perkin Elmer, Waltham, MA, USA), at 25°C, using an excitation wavelength of 440 nm and recording the ThT fluorescence emission spectra between 450 and 600 nm. The emission maximum, at 480 nm, was used to verify the presence of β-sheet enriched protein, comparing the fluorescence intensity of the emission peak to that of the monomeric protein in solution.

### TEM and Atomic Force Microscopy (AFM)

TEM fibril samples were prepared by adsorbing αSyn fibrils in PBS buffer at pH 7.4 (equivalent concentration about 25 µM) onto a carbon-coated copper grid and then performing negative staining with a 0.05% uranyl acetate solution. TEM images of αSyn fibrils were acquired to verify the morphology and dimensions of the fibrils used for the experiments (Fig. S1A and B).

Morphological characterization of monomeric and fibrillar αSyn was also performed, by AFM imaging (Fig. S2 and Text S1).

Monocytes were fixed overnight in 0.1 M sodium cacodylate buffer at pH 7.4 containing 2.5% glutaraldehyde and then processed and embedded in LR White resin (Polysciences, Warrington, PA, USA). Ultrathin sections were then stained with 2% uranyl acetate for TEM imaging.

TEM micrographs were taken on a Tecnai G2 12 Twin instrument (FEI Company, Hillsboro, OR, USA).

### Monocyte Isolation and Culture

PBMCs from healthy donors were isolated by centrifugation on Ficoll-Paque solution and laid on Percoll 46% v/v solution in RPMI 1640, 10% FCS and 4 mM HEPES. Monocytes were harvested, resuspended in RPMI 1640, 2% FCS and separated from contaminating lymphocytes by adherence to plastic (1 h at 37°C). Adherent monocytes were extensively washed with medium to remove residual non-adherent cells. Monocytes were then cultured in RPMI 1640, 1% Nutridoma-SP and stimulated with fibrillar αSyn (40 nM), monomeric αSyn (1 µM) or vehicle (PBS); when required, cells were pre-incubated for 30 min with Ac-YVAD-CMK (50 µM), DPI (20 µM), Baf A1 (250 nM), CA-074-Me (10 µM) and anti-TLR2 blocking antibody (20 µg/ml).

### Western Blot Analysis

Treated monocytes were collected, washed in ice-cold PBS, and lysed for 30 min at 4°C with ice-cold lysis buffer (20 mMTris, pH 7.15, 150 mM NaCl, 0.25% Nonidet P-40, supplemented with protease inhibitors: 1 mM PMSF, 1 µg/ml leupeptin, and 1 µg/ml aprotinin). Cell lysates were centrifuged at 12000*g* for 20 min at 4°C, the supernatants (cell extracts) were kept and the protein content was quantified using the BCA assay. The entire culture supernatants (600 µl) were collected, precipitated with 10% TCA for 1 h at room temperature; protein pellets were resuspended in 15 µl of lithium dodecyl sulfate, boiled, and conserved at −20°C. Two micrograms of cell extracts and the total protein content of culture supernatants were loaded on a 4–12% SDS-PAGE and analyzed by immunoblotting. Activated caspase-1 and IL-1β were revealed by specific antibodies. β-actin and albumin were used as control for equal loading of cell lysates and cell supernatants, respectively.

### Real-time PCR Analysis

Total RNA was extracted from 2×10^6^ monocytes using TRIzol solution, according to the manufacturer's instructions. RNA was reverse transcribed using SuperScript II and cDNA was amplified with the following primers: 5′-AGCAACAGGGTGGTGGAC-3′ and 5′-GTGTGGTGGGGGACTGAG-3′ for GAPDH; 5′-CTGTCCTGCGTGTTGAAAGA-3′ and 5′-TTGGGTAATTTTTGGGATCTACA-3′ for IL-1β; 5′-GGAGAGACCTTTATGAGAAAGCAA-3′ and 5′-GCTGTCTTCCTGGCATATCACA-3′ for NLRP3; 5′-GGAGGCCTTGGTGAAACC-3′ and 5′-CGATGTCACTCGGGCTATCA-3′ for NLRP1. After amplification, data analysis was performed using the second derivative method algorithm by applying the 2?^−ΔΔCT^ method [46]. For each sample, data were normalized to an endogenous reference gene (GAPDH). Cells harvested at time zero were taken as the reference value, set to 1 AU (arbitrary unit, as shown in the figures), and the relative expression levels for treated or untreated cells were calculated and shown.

### Detection of IL-1β in Culture Supernatants

Supernatants of monocyte cultures, harvested for quantification of mRNAs, were collected and stored at −80°C for subsequent quantification of cytokine content by IL-1β specific ELISA kit (eBioscience, San Diego, CA, USA), according to the manufacturer’s instructions.

### ROS Detection

Monocytes (2×10^6^ cells/well) were pre-incubated with 20 µM DPI, or left untreated, and then exposed to 40 nM αSyn fibrils (F), 1 µM monomer (M) or 1 µg/ml LPS for different periods. Cells were then incubated with the oxidant-sensitive probes 2′,7′-dichlorofluorescein diacetate (H_2_DCF-DA, 10 µM) in HBSS, glucose 10 mM for 45 min before being washed. Fluorescence was measured with a microplate fluorometer with excitation at 480 nm and emission at 530 nm.

### Confocal Microscopy

Monocytes (2×10^6^ cells/well in 24-well plates) were seeded on coverslips and treated with rhodamine-labelled 8 nM αSyn in absence or presence of Baf A1. Cells were then fixed for 10 min in cold methanol, nuclei were stained with Hoechst and the coverslips were mounted on glass slides. Cells were visualized with a 63× oil immersion objective on a laser-scanning confocal microscope and images were acquired using the LAS-AF software (Leica TCS-SP5, Leica Microsystems, Wetzlar, Germany).

### Statistical Analysis

Statistical analyses were performed using Student’s *t* test preceded by ANOVA and data, reported as the mean ± S.D., were considered significant if *p*-values ≤0.05.

## Results

### Fibrillar αSyn Induces IL-1β Synthesis and Release in Human Monocytes

It is known that αSyn activates microglial cells to produce pro-inflammatory cytokines, such as IL-1β [17]. To investigate the mechanism of the αSyn-triggered synthesis, maturation and release of IL-1β *in vitro*, we incubated primary human monocytes with fibrillar αSyn (αSyn F). Monocytes were preferred to macrophages or microglial cells, particularly for the aim of verifying whether αSyn acted also as transcriptional inducer, because of the open debate on the latter two cell types. There is evidence supporting the idea that macrophages or microglia have to be primed with LPS to ensure robust induction of pro-IL-1β [33], [34], [35], whereas other findings claim that LPS priming is not necessary to activate these cells [20].

**Figure 1:**
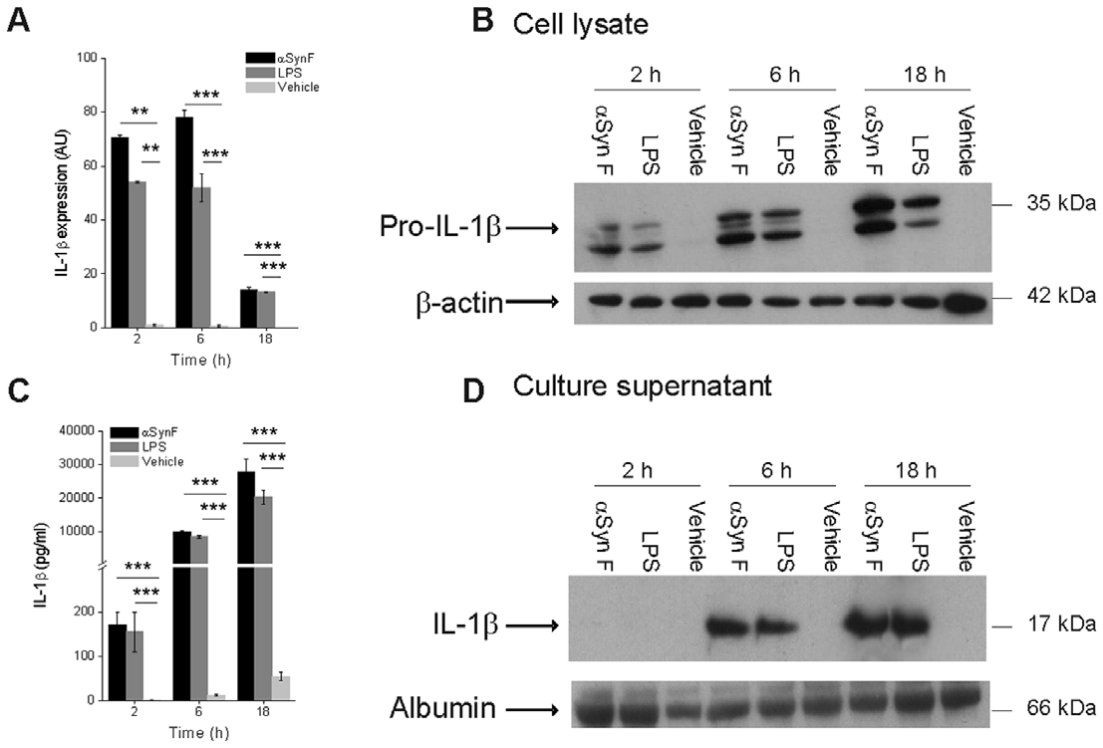
IL-1β synthesis and secretion in human monocytes induced by fibrillar αSyn. (A) Monocytes were exposed for 2, 6 and 18 h to 40 nM αSyn F or 1 µg/ml LPS (positive control) or vehicle (saline) and the expression of pro-IL-1β was evaluated by real-time PCR. Real time data are shown as the mean ± S.D. of results obtained with cell preparations from 5 different donors; experiments with each cell preparation were conducted in duplicate. **p<0.01, ***p<0.001. (B) IL-1β synthesis was also evaluated by immunoblot analysis in cell lysates from monocytes treated as in A. The immunoblot is from one representative donor. (C) Culture supernatants from monocytes that had been harvested for quantification of mRNA levels, reported in A, were collected and evaluated for their IL-1β content by ELISA. Results are the mean ± S.D. of 5 determinations made in duplicate with different cell preparations derived from different donors. ***p<0.001 (D) Culture supernatants of monocytes, harvested for the evaluation of IL-1β synthesis, as in B, were collected and the released IL-1β revealed in immunoblot.

We found that αSyn F rapidly induced the expression of pro-IL-1β to an extent comparable to that observed following LPS stimulation: this was detectable both at the level of mRNA (Fig. 1A) and protein (Fig. 1B). It is worth mentioning that, although in a fibrillar form, αSyn induced the transcription of the pro-IL-1β gene, while other particulate structures, such as silica needle-like crystals and monosodium urate (MSU) induced the secretion of the mature cytokine from LPS-primed monocytes, but not its synthesis [35], [38].

Finally, in addition to inducing pro-ILβ mRNA and protein, αSyn F induced also cleavage and release of the mature cytokine. IL-1β release was measured by enzyme-linked immunosorbent assay (ELISA; Fig. 1C) and it strongly correlated with the detected value of cleaved IL-1β by immunoblot analysis (Fig. 1D), indicating that activated cells released mature IL-1β.

### IL-1β Maturation Induced by αSyn F requires Caspase-1 Activation and Involves the NLRP3 Inflammasome

**Figure 2:**
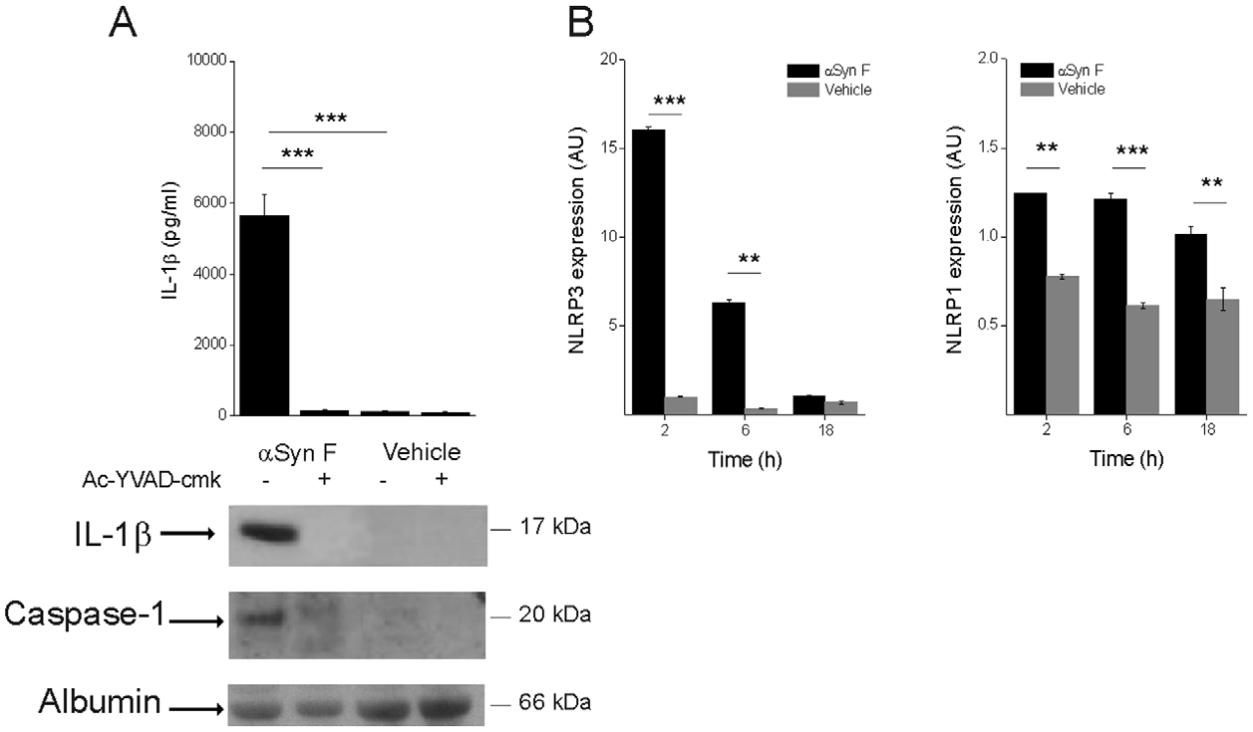
IL-1β induced by αSyn F is mediated by caspase-1 activation and involves NLRP3. (A) Monocytes were pre-incubated for 30 min with 50 µM Ac-YVAD-cmk, or left untreated, before being exposed for 6 h to 40 nM αSyn F or vehicle. IL-1β released into the culture supernatants was evaluated both by ELISA and immunoblot analysis. The same supernatants were assessed for the accumulation of active caspase-1 by immunoblot. Results are the mean ± S.D. of 3 experiments conducted in duplicate with cell preparations obtained from 3 different donors. The immunoblot is from one representative donor. ***p<0.001 (B) Real-time PCR of NLRP1 and NLRP3 in monocytes stimulated for 2, 6 and 18 h with αSyn F. Real time data are shown as the mean ± S.D. of results obtained with cell preparations obtained from 2 different donors; experiments with each cell preparation were conducted in duplicate. **p<0.01 and ***p<0.001.

IL-1β maturation is controlled by caspase-1 after assembly of the inflammasome, which activates pro-caspase-1. To test whether αSyn F activates caspase-1, we evaluated the release of both the active protease and IL-1β from monocytes pre-incubated with the caspase-1 specific inhibitor Ac-YVAD-cmk. Immunoblot analysis, performed with a polyclonal antibody against the active subunit p20 of caspase-1, revealed that the caspase inhibitor completely abolished the release of the mature form of the enzyme in response to the treatment with αSyn F. Moreover, the secretion of the mature cytokine was also impaired, proving that processing and secretion of IL-1β, induced by αSyn F, involves caspase-1 activation and, therefore, the inflammasome complex (Fig. 2A).

A recent body of data revealed that needle-like particulate material (*i.e.*, silica and MSU crystals, and fibrillar amyloid-β) activates the NLRP3 inflammasome [35], [38], [47]. Under resting conditions, NLRP3 is expressed but a pro-inflammatory signal is required to induce its expression to a level that leads to its activation [48]. Therefore, we decided to verify whether αSyn F increased the expression of NLRP3: in monocytes exposed to αSyn F, we measured the induction of mRNA encoding NLRP3 and compared it with that of NLRP1 (Fig. 2B). The results show that the protein induced a robust up-regulation of NLRP3, while the effect on NLRP1 expression was weaker. These data suggest that NLRP3 is the inflammasome mainly involved in αSyn F-induced IL-1β maturation.

### Phagocytosis of αSyn F is Required for IL-1β Release

**Figure 3:**
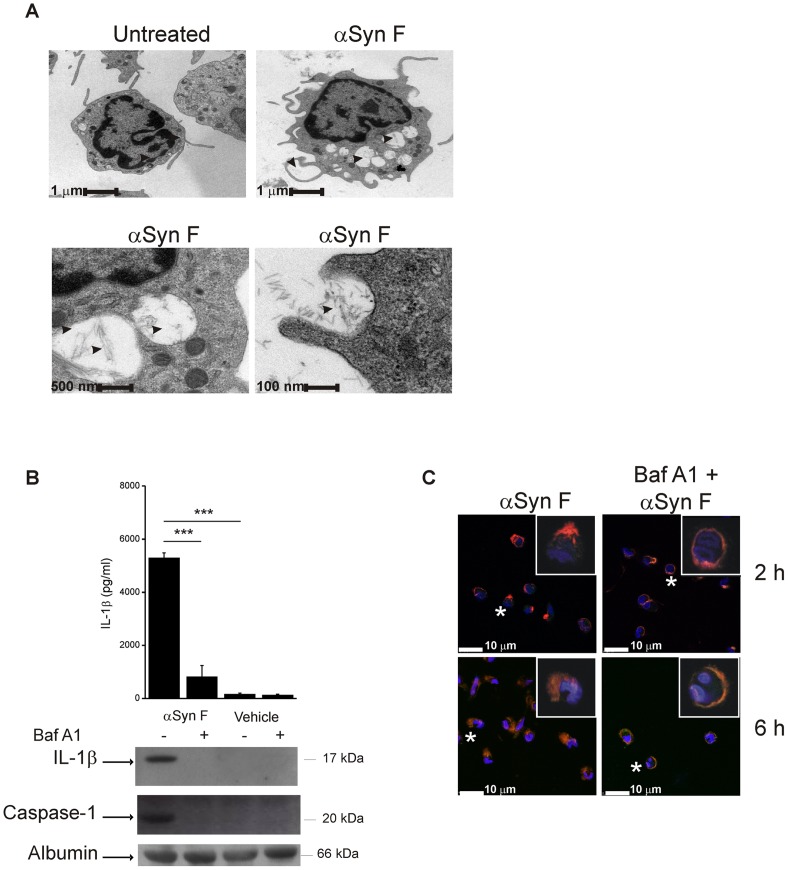
Phagocytosis of αSyn F is required for IL-1β release. (A) Transmission electron microscopy of monocytes treated for 6 h with 40 nM αSyn F, or left untreated. For αSyn F-treated cells, three magnifications are shown (bars represent 1 µm, 500 nm and 100 nm). Pictures of 20–30 cells for each condition were kept and 3 representative cells are shown. Left bottom panel is the magnification of two αSyn-containing vacuoles of the cell in the right upper panel. Arrowheads indicate the fibrils. (B) Monocytes were pre-incubated for 30 min with 250 nM Baf A1, or left untreated, before being exposed for 6 h to αSyn F or vehicle. IL-1β released into the culture supernatants was evaluated both by ELISA and immunoblot analysis. The same supernatants were assessed for the accumulation of active caspase-1 by immunoblot. Results are the mean ± S.D. of 3 experiments conducted in duplicate with cell preparations obtained from 3 different donors. The immunoblot is from one representative donor. ***p<0.001. (C) Confocal microscopy of monocytes incubated with 250 nMBaf A1 before and during the exposure for 2 or 6 h to αSyn F. Red staining corresponds to rhodamine-labelled αSyn F, while the Hoechst blue staining labels nuclei. White asterisks indicate the cells of which a magnification is shown (white square).

We next explored the mechanism by which αSyn F activates NLRP3 inflammasome. It is established that, in case of particulate material, inflammasome activation occurs following its phagocytosis [38], [47], [49]. Therefore, we first evaluated whether monocytes internalized the fibrils. Transmission electron microscopy (TEM) analyses of monocytes exposed to αSyn F for 6 h (a time considered sufficient to induce detectable IL-1β release) revealed that αSyn F accumulated in the cells in enlarged and swollen compartments, probably phagosomes or phago-lysosomes; this observation, together with the fact that no similar structures were appreciable in monocytes treated with saline (vehicle), demonstrated that αSyn F was phagocytosed by monocytes and that phago-lysosome swelling, and possible dysfunction, occurs during internalization (Fig. 3A). It is worth noting that, in TEM images, αSyn F can be clearly discriminated within these swollen compartments. We therefore investigated whether phagocytosis was required for αSyn F-induced release of IL-1β. We incubated monocytes, before and during stimulation with αSyn F, with Baf A1: this macrolide antibiotic neutralizes acidic organelles, such as endosomes, and inhibits the endocytotic pathway from early endosomes [50]. As expected, Baf A1 did not affect IL-1β synthesis induced by αSyn F (Fig. S3); however, it dramatically reduced αSyn F-mediated IL-1β release, as well as caspase-1 activation, as revealed by both ELISA and immunoblot analyses (Fig. 3B), and maintained confined αSyn F just below the plasma membrane (Fig. 3C). Collectively, these data show that αSyn Fis phagocytosed and that it accumulates in phagosomal compartments that become swollen, suggesting that a destabilization occurs; most importantly, internalization of the protein is essential for αSyn F-induced IL-1β release.

### Lysosomal Destabilization and ROS Production Trigger αSyn F-induced Inflammasome Activation

**Figure 4:**
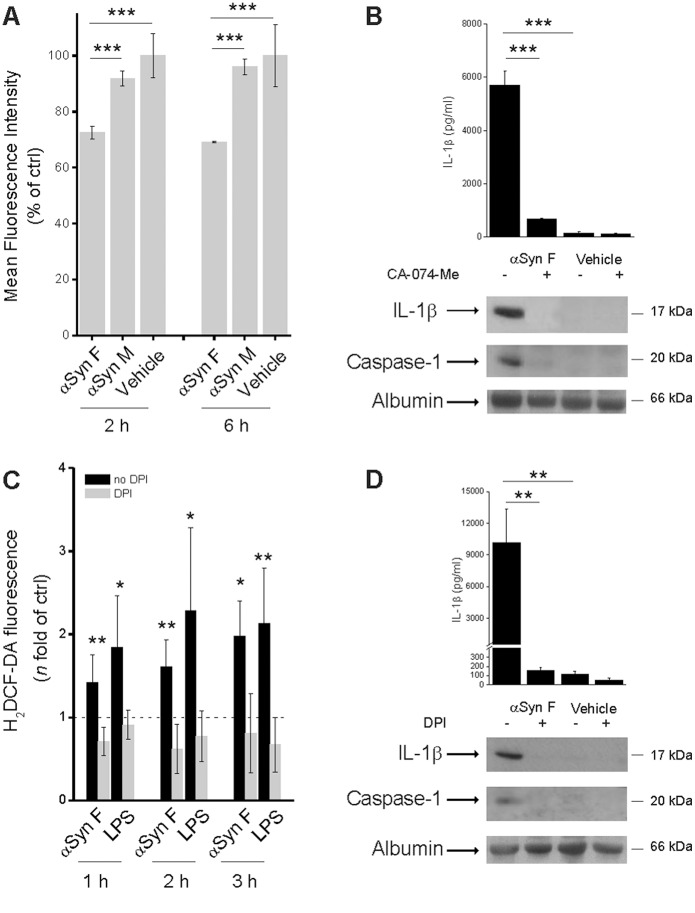
Cathepsin B activity as well as ROS production are involved in αSyn F-induced inflammasome activation. (A) Phagocytosis of fibrillar αSyn leads to phago-lysosomal destabilization. Flow cytometry of monocytes stained with acridine orange and then treated for 2 or 6 h with 40 nM αSyn F, 1 µM αSyn M. Data are reported as the percentage of acridine orange-positive cells present in the vehicle-exposed sample. Results are the mean ± S.D. of 3 experiments conducted in duplicate with cell preparations obtained from 3 different donors. ***p<0.001. (B) Monocytes were incubated for 30 min with 10 µM CA-074-Me (an inhibitor of cathepsin B), or left untreated; cells were then exposed for 6 h to 40 nM αSyn F or vehicle. IL-1β released into the culture supernatants was evaluated both by ELISA and immunoblot analysis. The same supernatants were assessed for the accumulation of active caspase-1 by immunoblot. Results are the mean ± S.D. of 3 experiments conducted in duplicate with cell preparations obtained from 3 different donors. The immunoblot is from one representative donor. ***p<0.001. (C) Monocytes were cultured for 1, 2 or 3 h in the presence of 1 µg/ml LPS or 40 nM αSyn F and intracellular ROS levels were quantified by the H_2_DCF-DA fluorometric method. When required, monocytes were pre-incubated for 30 min with 20 µM DPI. The dotted line refers to the basal levels of ROS production (as in untreated monocytes). Results are the mean ± S.D. of 5 experiments conducted in duplicate with cell preparations obtained from 5 different donors. *p<0.05, **p<0.01 was calculated for samples not exposed to DPI *vs* the correspondent samples with DPI. (D) Monocytes were pre-incubated for 30 min with 20 µM DPI, or left untreated, before being exposed for 6 h to αSyn F or vehicle. IL-1β, as well as activated caspase-1, were assessed as before. Results are the mean ± S.D. of 3 experiments conducted in duplicate with cell preparations obtained from 3 different donors. The immunoblot is from one representative donor. **p<0.01.

It is established that inflammasome activation, which follows phagocytosis of particulate substances, requires the release of active cathepsin B from lysosomes into the cytosol [35]. In order to prove that αSyn F destabilizes the phagosomal membrane, the pH-dependent fluorescence probe acridine orange was trapped within the phago-lysosomes and its leakage into the cytosol was monitored as fluorescence change. Cytofluorimetric analysis revealed that the monomeric protein was not competent to induce leakage of the probe, while αSyn F destabilized the phagosomal membrane already after a 2 h-treatment (Fig. 4A).

To investigate whether the activity of cathepsin B, after stimulation with αSyn F, was functionally linked to inflammasome activation, we measured the effect of the inhibition of cathepsin B on the release of IL-1β from monocytes. We found that the specific inhibitor of cathepsin B, CA-074-Me, strongly inhibited IL-1β release. Moreover, the fact that, in the presence of the inhibitor, the activation of caspase-1 was also impaired, demonstrated that the cathepsin B-induced release of IL-1β occurred through activation of caspase-1 and not independently (Fig. 4B).

Collectively, these data indicate that phagocytosis of αSyn F by monocytes leads to an enlargement of phago-lysosomes that is associated with a loss of their membrane integrity: the release of specific proteases, such as cathepsin B, into the cytosol is causally related to inflammasome activation.

Next, we assessed the requirement of ROS generation, which has been implicated in inflammasome activation in response to the majority of known NLRP3 agonists [51]. The exposure of monocytes to αSyn F resulted in a time-dependent increase of ROS production (Fig. 4C): notably, levels of ROS induced by αSyn F were comparable to those elicited by LPS, which is a known trigger of ROS production in immune cells [52]. Pre-treatment of monocytes with (DPI), a chemical inhibitor of ROS generation, diminished the amount of ROS (Fig. 4C) as well as inflammasome-dependent caspase-1 activation and IL-1β secretion in response to αSyn F (Fig. 4D).

Collectively, our data demonstrate that αSyn F-induced inflammasome activation requires phagocytosis, cathepsin-like activity and ROS production.

### The Activation of the Inflammasome is a Peculiarity of αSyn F, not Shared by its Monomeric Form

As mentioned above, αSyn exists in a monomeric form but, under particular conditions, it assembles into aggregates which are thought to have a pathogenic role in PD due to their pro-inflammatory activity [17], [53]. Considering that we could not exclude that also monomers were endowed with pro-inflammatory properties, we addressed the role of monomeric αSyn (αSyn M) in promoting the synthesis of pro-IL-1β and in activating the inflammasome. The concentration of monomer we adopted for the experiments was 1 µM, while the monomer equivalent molar concentration for αSyn fibrils was about 5 µM. However, the concentration of monomers is likely to be much higher than the actual concentration of αSyn F used in the experiments described above, because the formation of an individual αSyn fibrils is likely to recruit thousands of monomers. Therefore, the actual concentration of αSyn F used is expected to be in the range of nM, much lower than the concentration of αSyn M (1 µM).

**Figure 5:**
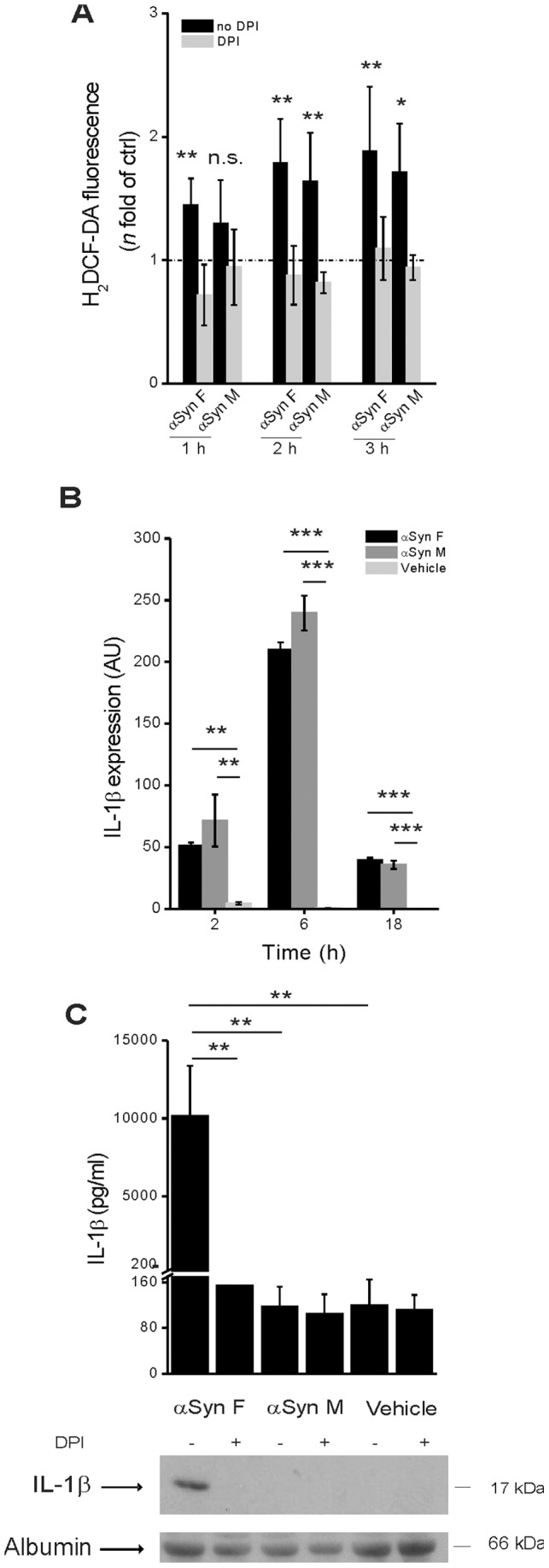
Only αSyn F is capable of activating the inflammasome. (A) Monocytes were cultured for 1, 2 or 3 h in the presence of 40 nM αSyn F or 1 µM αSyn M and intracellular ROS levels were quantified by the H_2_DCF-DA fluorometric method. When required, monocytes were pre-incubated for 30 min with 20 µM DPI. The dotted line refers to the basal levels of ROS production (as in untreated monocytes). Results are the mean ± S.D. of 5 experiments conducted in duplicate with cell preparations obtained from 5 different donors. *p<0.05, **p<0.01 was calculated for samples not exposedto DPI *vs* the correspondent samples with DPI. (B) Monocytes were exposed for 2, 6 and 18 h to 40 nM αSyn F, or 1 µM αSyn M or vehicle (saline) and the expression of pro-IL-1β was evaluated by real-time PCR. Real time data are shown as the mean ± S.D. of results obtained with cell preparations from 2 different donors; experiments with each cell preparation were conducted in duplicate. **p<0.01 and ***p<0.001. (C) Monocytes were pre-incubated for 30 min with 20 µM DPI, or left untreated, before being exposed for 6 h to αSyn F or αSyn M or vehicle. IL-1β was determined as before. Results are the mean ± S.D. of 3 experiments conducted in duplicate with cell preparations obtained from 3 different donors. The immunoblot is from one representative donor. **p<0.01.

We first compared the ability of the two forms of protein to induce ROS production; interestingly, both αSyn M and αSyn F induced ROS production in monocytes to a similar extent and with a similar time dependence. Moreover, in both cases, pre-incubation of cells with DPI strongly reduced ROS production (Fig. 5A).

Analysis of pro-IL-1β mRNA expression revealed that it was similarly induced by αSyn M and αSyn F (Fig. 5B); on the contrary, analysis of culture supernatants revealed that the monomeric αSyn was unable to induce the release of mature IL-1β (Fig. 5C). These results suggest that ROS are necessary but not sufficient for αSyn-induced inflammasome activation and that lysosome destabilization, induced only by fibrils, is a required factor for IL-1β maturation.

### TLR2 is Involved in IL-1β Expression Induced by αSyn

**Figure 6:**
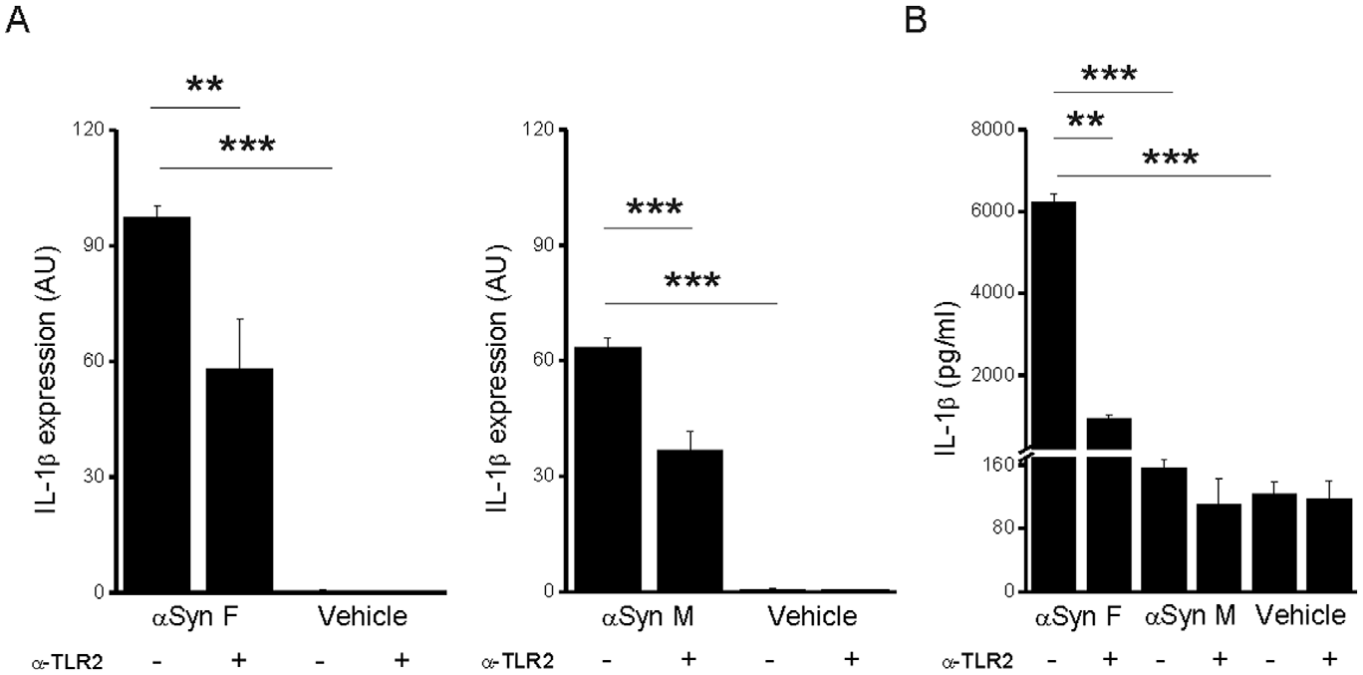
TLR2 engagement is involved in IL-1β induction by αSyn. (A) Levels of pro-IL-1β mRNA in monocytes were determined by quantitative real-time PCR analysis after a 6 h stimulation with αSyn F or αSyn M in the presence or not of a soluble anti-TLR2 antibody. Real time data are shown as the mean ± S.D. of results obtained with 2 cell preparations obtained from 2 different donors; experiments with each cell preparation was conducted in duplicate. (B) IL-1β content in the culture supernatant of the same cells harvested for mRNA evaluation was determined by ELISA. Results are the mean ± S.D. of 2 experiments conducted in duplicate with cell preparations obtained from 2 different donors. **p<0.01 and ***p<0.001.

Microglia cells express on their surface Toll-like receptors (TLRs); these receptors recognize conserved molecular motifs associated to pathogens (PAMPs) or endogenous danger signals (DAMPs). The engagement of a TLR triggers a downstream molecular pathway ending with the translocation of the transcription factor NFκB to the nucleus and the subsequent up-regulation of the expression of pro-inflammatory molecules [54]. It has been recently reported that αSyn activates this pathway and also that it modulates TLR expression: in particular, it up-regulates the expression of TLR2 [16]. Therefore, the possibility is there that ®Syn binds to TLR2. To address such a possibility, monocytes were pre-incubated with a TLR2-blocking antibody before stimulation with αSyn F or αSyn M. Real-time PCR analysis revealed that, in presence of the blocking antibody, the expression of pro-IL-1β was strongly reduced, independently of the form of the protein (Fig. 6A). These data show that both fibrillar and monomeric αSyn are able to activate an inflammatory response and demonstrate that the latter involves the interaction with TLR2. As a consequence of the reduced synthesis of IL-1β, also the level of the mature cytokine decreased. The effect of the blocking antibody on the release of the mature cytokine induced by αSyn M was negligible: this is expected since it was also negligible the amount of cytokine released from monocytes exposed to the monomeric form in absence of any antibody (Fig. 5 and Fig. 6B).

## Discussion

The neurodegenerative process in PD and related synucleinopathies is accompanied by the presence of a neuro-inflammatory response, which has been proposed to contribute to disease progression [55], [56]. Several studies lead to the definition of a role for αSyn in the initiation and maintenance of inflammation in PD, through the activation of microglia cells [57], [58]. Once activated, these cells would release the pro-inflammatory TNF-α and IL-1β cytokines [17], [56], thus leading to the accumulation of reactive oxygen species and generating an adverse environment for adjacent neurons, responsible for their damage and death [24], [59]. This is particularly true for dopaminergic neurons, in which the redox chemistry of dopamine present in the cytoplasm could be enhanced by ROS, leading to the formation of toxic dopamine-derived quinones [60].

Although it is established that αSyn may activate microglia, the mechanism by which this activation occurs remained an elusive issue. Within this framework, since αSyn is a natively unstructured protein that can present itself in several conformations, the definition of the form of αSyn which mainly exerts a pro-inflammatory activity becomes a germane question.

Here we demonstrated that insoluble αSyn fibrils induced monocytes to release IL-1β following the activation of NLRP3 inflammasome. In contrast to all previous studies, aimed at evaluating the pro-inflammatory activity of αSyn and using microglia as a cell model [16], [56], we made the choice to work with monocytes. Our choice is based on two reasons. First, several reports have demonstrated that macrophage-like cells, such as microglia, have to be primed with LPS to ensure robust induction of pro-IL-1β [33], [34], [35]; this experimental approach not only hampers the possibility of verifying whether an agonist promotes by itself the expression of the cytokine but, most importantly, it does not replicate the *in vivo* situation of PD patients, in whose brains LPS is not present. The second reason relied on the evidence that blood circulating monocytes are recruited during inflammatory events occurring in the central nervous system and they are responsible for microglial cell replenishment [61], [62]; therefore, they may reasonably represent a target of αSyn *in vivo*.

When we exposed monocytes to fibrillar αSyn, we found that not only it stimulated the release of the mature cytokine IL-1β but, notably, it also enhanced its gene expression. This result differentiates αSyn from other particulate structures that induce the secretion of IL-1β activating inflammasome, without inducing its synthesis [38], [47].

Our data support the view that IL-1β synthesis occurs following the engagement of TLR2, which mediates a downstream pathway that results in the translocation of NFκB to the nucleus. Interestingly, αSyn engaged TLR2 and activated the downstream pathway also as monomer. Although this observation sounds peculiar considering that monomers and fibrillar αSyn are structurally very different, it must be considered that TLR2 has the capability to recognize a wide range of structurally unrelated PAMPs and DAMPs [63]. However, since the C-terminal domain is unstructured and solvent-exposed in both monomeric and fibrillar αSyn, the possibility exists that the interaction between TLR2 and αSyn occurs within this region [64].

The similarities between αSyn F and αSyn M are restricted to their ability to induce the expression of pro-IL-1β. It is only the former that is capable of inducing the secretion of the mature cytokine, following the activation of inflammasome. We showed by confocal microscopy and TEM that monocytes phagocytosed αSyn fibrils that, accumulating in phago-lysosomes, led to their swelling. Phagocyotosis of αSyn F is crucial for its ability to activate the inflammasome, as demonstrated by the fact that once phagocytosis was hampered with Baf A1 in monocytes, the secretion of IL-1β induced by αSyn F was markedly blocked.

The leakage of the lysosomal cysteine protease cathepsin B into the cytosol has been associated with the activation of the NLRP3 inflammasome by silica and cholesterol crystals [35], [65] and it reflects a destabilization of the membrane of phago-lysosomes as a consequence of their swelling. Similarly, also for αSyn the cytosolic activity of cathepsin B seemed to be an essential intermediate step for inflammasome activation, indicating that fibrils-induced swelling of phago-lysosomal compartments resulted in an alteration of their membrane integrity and in the leakage of the enzyme into the cytosol.

Finally, in accordance with the fact that phagocytosis triggers the assembly of the NADPH oxidase complex on the plasma membrane and on the phagosomal membrane, we found that αSyn induces the production of oxygen radicals, which are also crucial for inflammasome activation. Fitting together all these data, allows us to propose a model outlining how αSyn may exert its pro-inflammatory activity.

Moreover, the characterization of the steps and molecules involved opens the possibility of reducing αSyn-triggered inflammation by blocking the activity of cytokine IL-1β, the final product of inflammasome activation. Albeit for a different disease, this blockage has already been pursued effectively: since 2002, one of the approaches for treatment of rheumatoid arthritis is the administration of Anakinra, an IL-1β receptor antagonist. It may therefore be worthwhile to explore the possibility of using an already FDA-approved drug as a new therapeutic avenue in PD, at least in managing the progression of the disease where neurodegeneration leads to the release of aggregated αSyn [15]. However, this potential approach has to be balanced with the possibility that turning off inflammation may enhance the neuron-to-neuron progression of the disease [66] because of the expected accumulation of extracellular αSyn F.

## Supporting Information

Figure S1 (TIF)

Figure S2 (TIF)

Figure S3 (TIF)

Text S1 (DOCX)
