# Supplementary figures and images for: Triggering of Inflammasome by Aggregated α–Synuclein, an Inflammatory Response in Synucleinopathies
Source: PLoS One. 2013 Jan 31;8(1):e55375. doi: 10.1371/journal.pone.0055375 (PMC3561263; doi:10.1371/journal.pone.0055375)

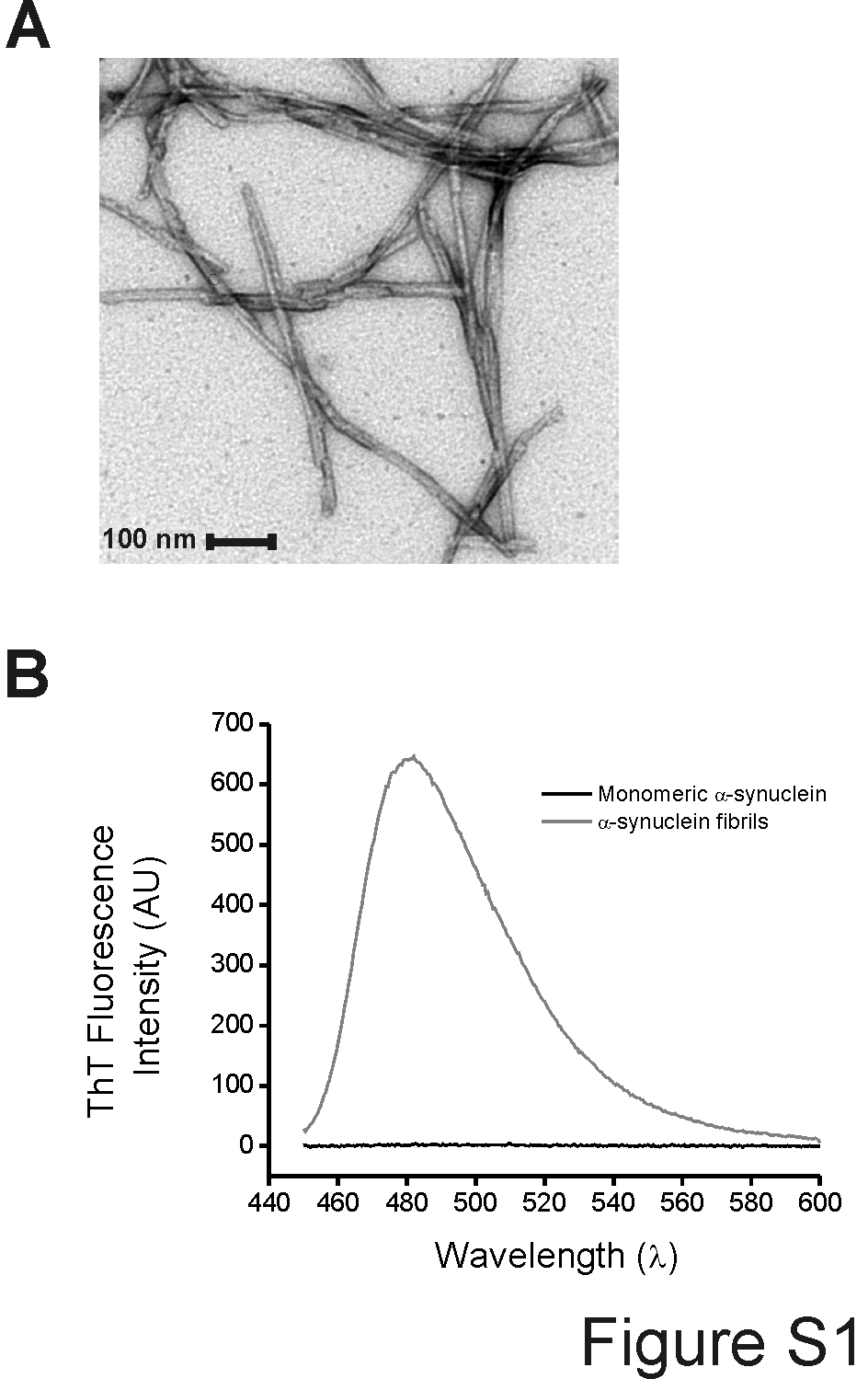

Supplement: Figure S1 — (TIF) [file pone.0055375.s001.tif]

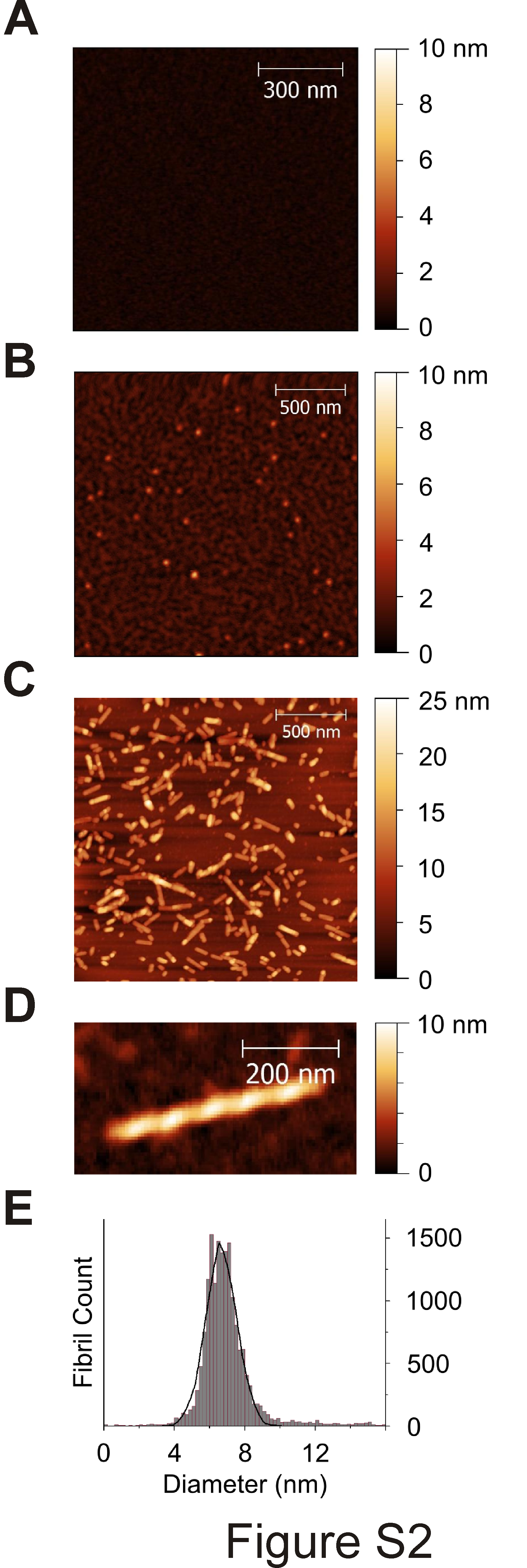

Supplement: Figure S2 — (TIF) [file pone.0055375.s002.tif]

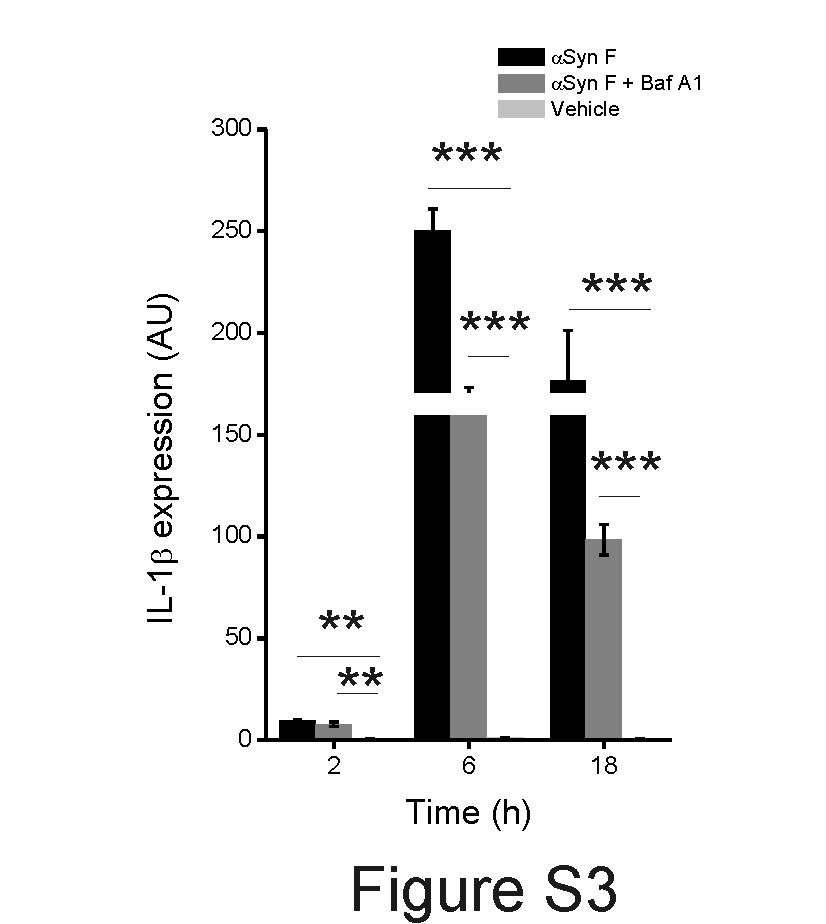

Supplement: Figure S3 — (TIF) [file pone.0055375.s003.tif]
