# Supplementary material for: Triggering of Inflammasome by Aggregated α–Synuclein, an Inflammatory Response in Synucleinopathies
Source: PLoS One. 2013 Jan 31;8(1):e55375. doi: 10.1371/journal.pone.0055375 (PMC3561263; doi:10.1371/journal.pone.0055375)
Supplement: Text S1 — (DOCX) [file pone.0055375.s004.docx]

**Method S1**

**Atomic Force Microscopy (AFM)**

For the morphological characterization of αSyn M and αSyn F, samples were diluted 1:20 to obtain an optimal concentration for AFM imaging. For the characterization of αSyn oligomeric intermediates by AFM, 10 μl aliquots from the aggregation mixture were taken after 6h of incubation and diluted in 90 μl of PBS, to be representative of the smallest detectable oligomers. Diluted samples were left to equilibrate at RT for 10 min, then deposited on freshly cleaved mica (RubyRed Mica Sheets, Electron Microscopy Sciences, Fort Washington, Maryland, USA) and left to adsorb for 2 min at RT (~20°C). The mica surface was then rinsed with ~500 μl of Milli-Q water at the same temperature and gently dried with dry nitrogen. Atomic force microscopy imaging was performed in tapping mode with NSC15 phosphorous-doped silicon probes (MikroMasch, Tallin, Estonia) on a NanoScopeIIIa system equipped with a Multimode head and a type E piezoelectric scanner (Veeco, Santa Barbara, CA, USA). Raw SFM images were processed only for background removal (flattening) by using Gwyddion v2.26 (<http://gwyddion.net/>). Diameters of the amyloid aggregates were measured from the AFM images via a semi-automated tool [1] that automatically recognizes fibrils and interpolates their shape with a cubic B-spline function. The Z scale values of the individual pixels crossed by the B-spline in the AFM image are pooled to obtain the distributions of the apparent diameters. When recording AFM images to be measured with the fibrils recognition tool, special care was taken to obtain images in which the fibrils appeared well spread and isolated on the surface rather than overlapped and tangled.

**References S1**

[1] Aioanei D (2012) Lazy Shortest Path Computation in DynamicGraphs. Computer Science 13:113-117.

**Results S1**

AFM images of monomers, oligomers and fibrillar aggregates of repeated incubation batches revealed an extremely reproducible pattern. Monomer aliquots showed the presence of copious amounts of amorphous material, but a distinct lack of identifiable aggregates. Sporadically, small globular objects were observed, which were also present in the aggregation buffer solution. Although individual αSyn monomers cannot be resolved in AFM images (Fig. S2A), local height maxima never exceeded 1.5 nm, thus ruling out the possibility that αSyn aggregates are present in solution at the start of the amyloidogenic process. While aliquots taken after 6h of incubation were in some batches indistinguishable from that ones taken at 0h, in most batches they showed the appearance of small globular aggregates having an apparent diameter of 2-8 nm (Fig. S2B). The distributions of apparent diameters were broad and non-Gaussian, suggesting that several different types of oligomeric aggregates coexisted at this stage of aggregation, the relative amount of which was extremely variable from batch to batch. However, these aggregates were easily distinguishable both from monomeric αSyn and mature fibrillar aggregates.The first fibrillar products appeared in some aggregation batches after as little as 12h of incubation, and aliquots taken after that time of incubation invariably showed the presence of large amounts of those and the total absence of globular oligomers (Fig. S2C). Their univocal identification was easy as they showed a left-handed helical periodicity of around 80 nm (Fig. 2D), while their measured diameters clustered at a value of 7.3 ± 0.8 nm, as shown by the Gaussian fit representing αSyn fibrils diameters distribution (Fig. S2E).

**Figure S1. Characterization of fibrillar αSyn.** (A) TEM image of αSyn fibrils. Fibrils used in all the experiments present the typical morphological features of αSyn fibrillar species, being micrometers long and having a mean diameter of about 8 nm. (B) ThT fluorescence spectra are shown for monomeric and fibrillar αSyn. The strong increase in ThT fluorescence intensity reflects an increase in β-sheet content, which characterizes fibrillar αSyn.

**Figure S2. Morphological characterization of αSyn aggregation products at different stages of the amyloidogenesis process.** (A) unaggregated monomer, (B) first identifiable globular oligomers, (C) mature fibrillar aggregates, (D) isolated fibril showing helicity and periodicity, (E) distribution of the apparent diameters of the fibrillar aggregates, as measured from AFM images *via* a semi-interactive software tool with superimposed Gaussian fit (solid black line).

**Figure S3. IL-1β synthesis is not affected by bafilomycin A1.** Monocytes were pre-incubated for 30 min with 250 nM Baf A1, or left untreated, before being exposed for 2, 6 or 18 h to αSyn F or vehicle. The expression of pro-IL-1β was evaluated by real-time PCR. Real time data are shown as the mean ± S.D. of results obtained with cell preparations from 2 different donors; experiments with each cell preparation were conducted in duplicate. ***p*<0.01, ****p*<0.001.
